# The impact of high-density atrioventricular dual-chamber mapping in a patient with a left epicardial accessory pathway

**DOI:** 10.1016/j.hrcr.2020.08.023

**Published:** 2020-09-11

**Authors:** Hitoshi Mori, Naokata Sumitomo, Shota Muraji, Noriyuki Iwashita

**Affiliations:** Department of Pediatric Cardiology, Saitama Medical University International Medical Center, Hidaka, Japan

**Keywords:** Accessory pathway, Atrioventricular dual-chamber mapping, Catheter ablation, Epicardial connection, Ultra-high-density mapping

## Introduction

Accessory pathways (APs) are one of the most common causes of supraventricular tachycardia, usually exist on the endocardium, and are located around the atrioventricular valves. Connections between the coronary sinus (CS) and ventricle have been reported previously, ablation of which can be successful only within the CS.^1^^,^^2^ However, to the best of our knowledge, the detailed conduction pattern of left epicardial APs using ultra-high-density mapping has never been reported.

**Key Teaching Points**

• When recurrence of conduction is frequently observed during endocardial accessory pathway ablation, you might consider the possibility of an epicardial connection.
• Posteroseptal accessory pathways have been recognized as the most frequent type of epicardial connections.
• Some epicardial accessory pathways can be ablated from the coronary sinus.
• This is the first report to demonstrate the detailed conduction pattern of a left epicardial accessory pathway using a high-density atrioventricular dual-chamber map.

## Case report

**Figure 1:**
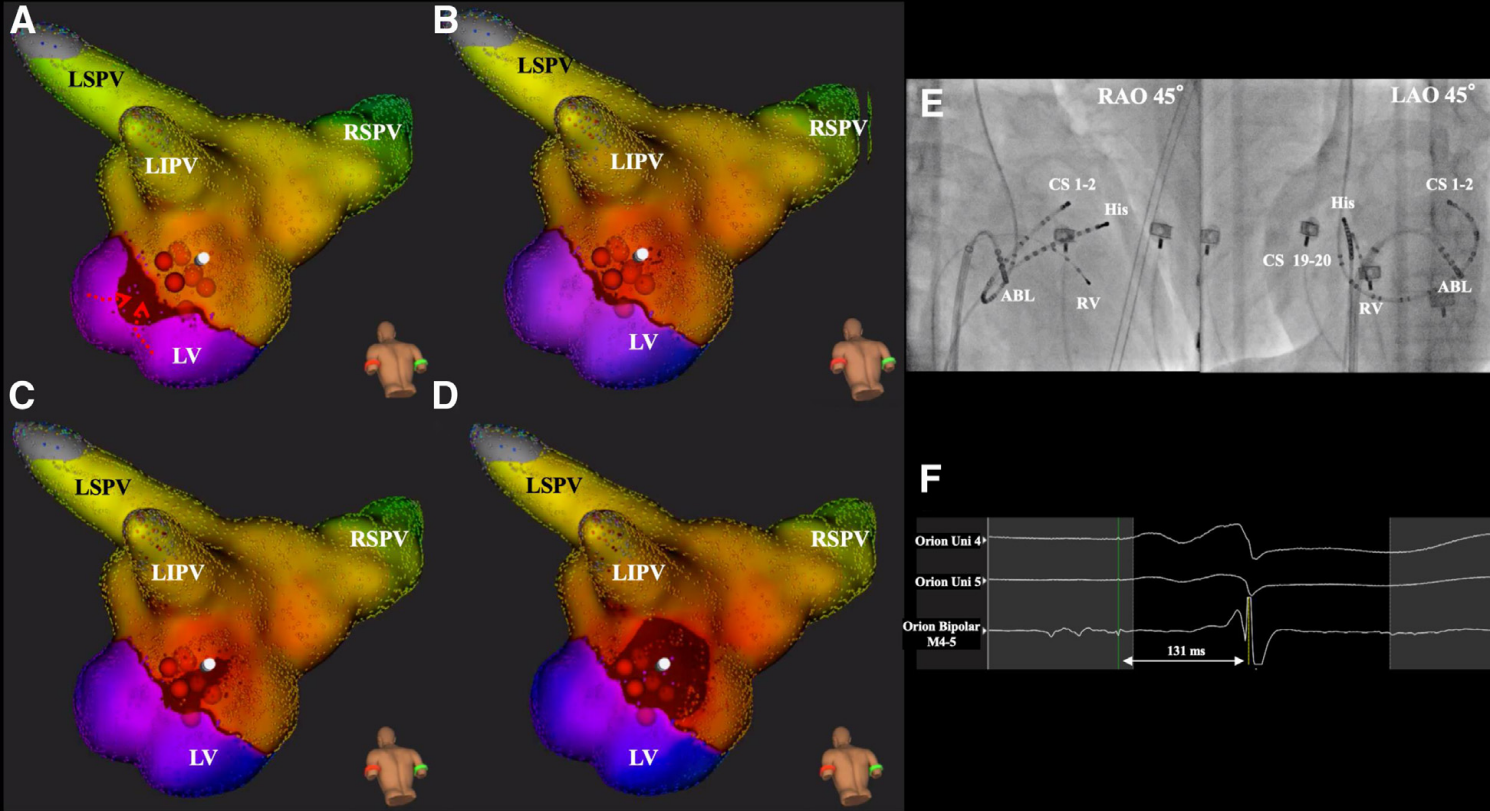
**A–D:** Dual chamber maps and activation pattern of the retrograde conduction via an accessory pathway from the endocardium. The deep red color shows the earliest activation site. The wavefront moved from the left ventricle to the left atrium (LA) and the Roving Probe shows the earliest atrial activation site (EAAS). The ablation energy (30W, 70 sec) was delivered at the EAAS from inside the LA, and the antegrade conduction and retrograde conduction terminated within a few seconds, however, the retrograde conduction recurred immediately after the discontinuation of the energy delivery (*red tag*). **E:** Fluoroscopic image of the ablation site. **F:** shows the local potential recorded from an Orion catheter at the EAAS in the LA, and the duration from the right ventricular pacing site to the local potential was 131ms (A-D, Roving Probe site). LIPV = left inferior pulmonary vein; LSVP = left superior pulmonary vein; RSPV = right superior pulmonary vein; CS = coronary sinus catheter; His = His bundle recording catheter; RV = right ventricle catheter; ABL = ablation catheter; RAO = right anterior oblique view; LAO = left anterior oblique view.

**Figure 2:**
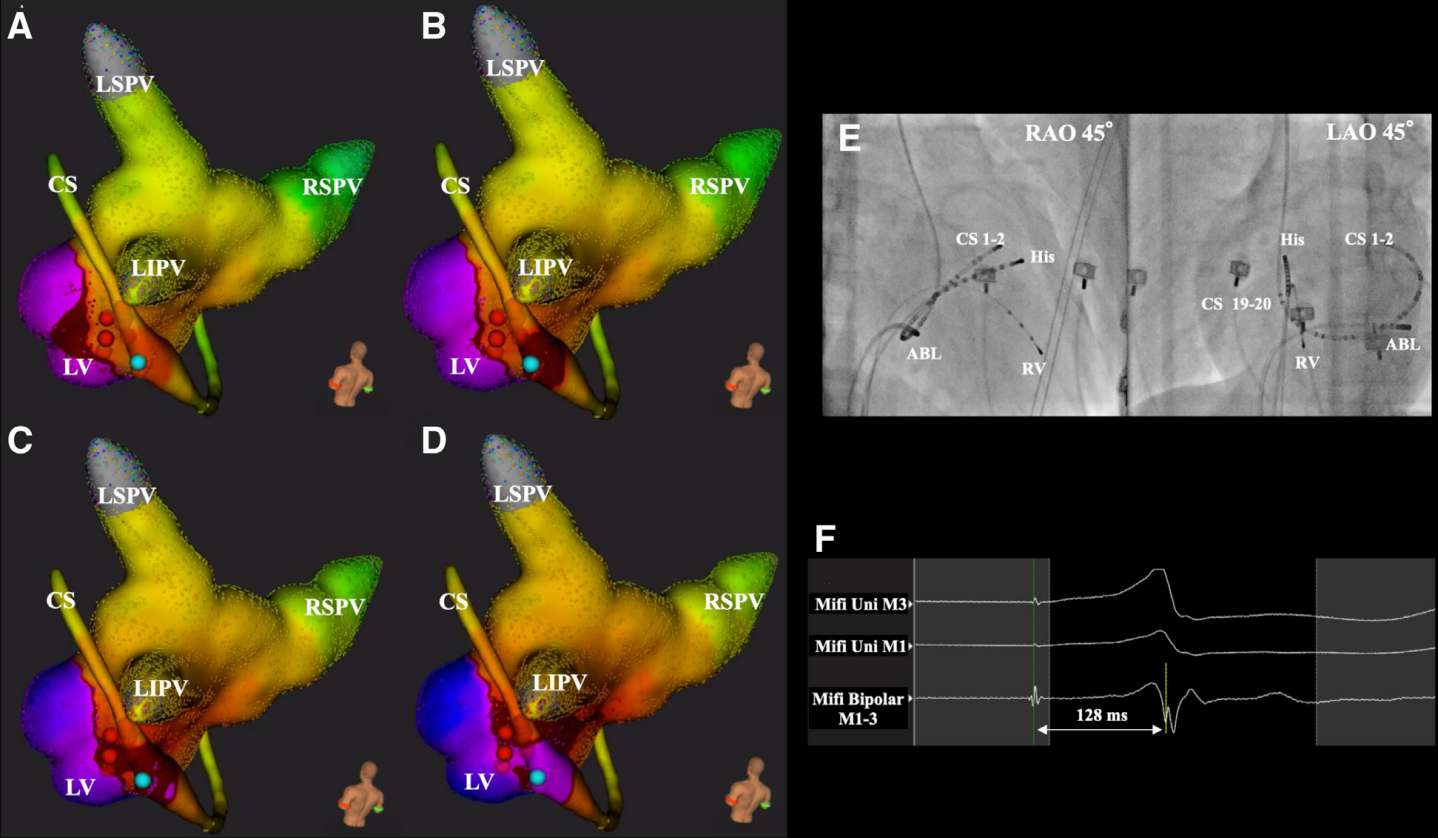
**A–D:** The activation pattern of the retrograde conduction via an accessory pathway from the coronary sinus. The red tag represents the ablation site from the left atrium (LA). The deep red color also represents the earliest activation site. The retrograde conduction spread from the left ventricle (LV) to the coronary sinus (CS) and then to the LA (A-D). The ablation energy (20W, 30 sec) was delivered from the CS (A-D, *blue tag*), and soon after the energy delivery inside the CS, the retrograde conduction was interrupted. **E:** The fluoroscopic image of the ablation site. **F:** The local potential recorded from the ablation catheter at the earliest activation site in the coronary sinus, and the duration from the right ventricular pacing site to the local potential was 128ms (B, blue tag site). LIPV = left inferior pulmonary vein; LSVP = left superior pulmonary vein; RSPV = right superior pulmonary vein; CS = coronary sinus catheter; His = His bundle recording catheter; RV = right ventricle catheter; ABL = ablation catheter; RAO = right anterior oblique view; LAO = left anterior oblique view.

A 15-year-old male patient was referred to our hospital for radiofrequency catheter ablation of a supraventricular tachycardia. His 12-lead electrocardiogram suggested left-sided Wolff-Parkinson-White syndrome. The earliest atrial activation site (EAAS) during ventricular pacing recorded by an ultra-high-density mapping system (Rhythmia; Boston Scientific, Marlborough, MA) was 6 o’clock on the mitral annulus (Figure 1A–D), which did not exhibit any decremental properties, and the conduction was not interrupted by an adenosine triphosphate infusion. The duration from the right ventricular pacing site to the local potential was 131 ms (Figure 1E and F). The ablation energy (30 W, 70 s) was delivered at the EAAS from inside the left atrium (LA), and the antegrade conduction and retrograde conduction terminated within a few seconds; however, the retrograde conduction recurred immediately after the discontinuation of the energy deliveries (Figure 1A–D, red tag). Several ablation attempts were tried from the LA, and the retrograde conduction always terminated within a few seconds; however, it recurred immediately after discontinuation of the energy deliveries. Although it would have a higher risk, we tried to map and ablate from the CS. CS mapping with a CS catheter and ablation catheter (IntellaNav; Boston Scientific, Marlborough, MA) (Figure 1 and Supplementary Video 1) revealed that the retrograde conduction spread from the left ventricle to the CS and then to the LA (Figure 2A–D and Supplementary Material). CS venography did not reveal any anatomical anomalies such as a diverticulum in the CS. The duration from the right ventricular pacing site to the local potential was 128 ms (Figure 2E and F). We lowered the ablation energy to 20 W and 30 s, and soon after the energy delivery inside the CS, the retrograde conduction was interrupted (Figure 2, blue tag).

The previous mapping systems make a point-to-point annotation of the earliest potential for the 3-dimensional activation maps. Considering that functional limitation, those systems could annotate only atrial or ventricular potentials and are not able to distinguish between atrial and ventricular potentials during the same mapping. The Rhythmia system has an intelligent annotation technology and annotates the largest bipolar potential, which enables distinguishing the atrial and ventricular potentials all at once and can construct an atrioventricular dual-chamber map without a manual reannotation.

## Discussion

Posteroseptal APs have been recognized as the most frequent epicardial APs, and successful ablation from inside the CS has been reported.^2^ However, to the best of our knowledge, this is the first report to demonstrate the detailed connection between the left ventricle and an epicardial AP. Supraventricular arrhythmia ablation using a 3-dimensional map system is useful for reducing the radiation exposure in young patients.^3^ It was also useful for helping us to understand the details of the connection of the AP in our case.
