# Supplementary material for: The impact of high-density atrioventricular dual-chamber mapping in a patient with a left epicardial accessory pathway
Source: HeartRhythm Case Rep. 2020 Sep 11;6(11):888–90. doi: 10.1016/j.hrcr.2020.08.023 (PMC7653477; doi:10.1016/j.hrcr.2020.08.023)
Supplement: Supplementary Material [file mmc2.docx]

**Supplemental File 1**

Propagation map of the retrograde conduction under right ventricular pacing. The retrograde conduction spread from the left ventricle to the coronary sinus (CS) and then to the left atrium (LA). The retrograde conduction was interrupted within a few seconds after the delivery of radiofrequency energy from the LA, however, the retrograde conduction recurred immediately after the discontinuation of the energy delivery (red tags). An ablation energy delivery at the site noted by the blue tag from the CS immediately and completely blocked the retrograde conduction. No further conduction recurred after the last energy delivery.
